# Potential of hydroethanolic leaf extract of *Ocimum sanctum* in ameliorating redox status and lung injury in COPD: an in vivo and in silico study

**DOI:** 10.1038/s41598-023-27543-1

**Published:** 2023-01-20

**Authors:** Atul Srivastava, Subhashini, Vinita Pandey, Vandana Yadav, Sangita Singh, Ragini Srivastava

**Affiliations:** 1 Department of Biochemistry, Institute of Medical Sciences, Banaras Hindu University, Varanasi, Uttar Pradesh 221005 India; 2 Neuroimmunobiology Lab, Department of Zoology, MahilaMahavidyalaya, Banaras Hindu University, Varanasi, Uttar Pradesh 221005 India

**Keywords:** Computational biology and bioinformatics, Immunology, Physiology, Health care, Medical research

## Abstract

Oxidative stress and inflammation are hypothesised as the main contributor for Chronic Obstructive Pulmonary Disease (COPD). Cigarette smoke (CS), a major cause of COPD leads to inflammation resulting in recruitment of neutrophils and macrophages which are rich sources of oxidants. Activation of these cells produces excess oxidants and depletes antioxidants resulting in stress. Presently, effective drug for COPD is limited; therefore, novel compounds from natural sources, including plants are under exploration. The present study aims to investigate the protective effect of *Ocimum sanctum* leaf extract (OLE) in CS − induced model of COPD. Exposure to CS was performed thrice a week for 8 weeks and OLE (200 mg/kg and 400 mg/kg) was administered an hour before CS exposure. Control group (negative control) were exposed to ambient air while COPD group was exposed to CS (positive control). Administration of OLE doses reduced inflammation, decreased oxidant concentration and increased antioxidant concentration (p < 0.01). Molecular docking studies between the major phytocompounds of OLE (Eugenol, Cyclohexane and Caryophyllene) and antioxidant enzymes Superoxide dismutase (SOD), Catalase, Glutathione peroxidase (GPx), Glutathione reductase (GR) and Glutathione S Transferase (GST) showed strong binding interaction in terms of binding energy. In vivo and in silico findings for the first time indicates that OLE extract significantly alleviates oxidative stress by its potent free radical scavenging property and strong interaction with antioxidant enzymes. OLE extract may prove to be a therapeutic option for COPD prevention and treatment.

## Introduction

Chronic obstructive pulmonary disease (COPD) has been recognized as a third leading cause of death globally^1^. It is well characterized by persistent respiratory symptoms including poorly reversible airflow limitation and obstruction and an inconsistent and dysregulated inflammatory reaction in the airways^2^. It has been evident that among the several factors contributing to the development and progression of COPD, the most commonly encountered risk factor is cigarette smoke (CS) which introduces high level of Reactive Oxygen Species (ROS) into the airways^3^. Chronic bronchitis, emphysema and destruction of the alveolar or parenchymal wall are also important pathophysiology of COPD^4^. Exposure to CS triggers lung epithelial cell damage and further infiltration and activation of macrophages and neutrophils lead to inflammatory response^2^. Both neutrophils and macrophages are rich sources of endogenous ROS and Reactive Nitrogen Species (RNS) which exert oxidant burden on the lungs facilitating alveolar damage^5^. The excess oxidant leads to the imbalance of oxidant and antioxidant leading to pulmonary oxidative stress^6^.

There is overwhelming evidence that oxidative stress leading to oxidative damage plays an elementary role in the pathophysiology of COPD and has important implications on several events of lung physiology. This influences antiproteases activity, surfactants, mucus secretion, membrane lipid peroxidation, alveolar epithelial injury, remodelling of extracellular matrix and apoptosis. Oxidative stress induced by accumulation of ROS including superoxide anion (O_2_), hydroxyl radical (OH), hydrogen peroxide (H_2_O_2_) and nitric oxide (NO) radicals or dysfunctioning of endogenous antioxidant defence system is a major driving mechanism underlying CS−induced COPD^6^. The endogenous antioxidant system comprises both enzymatic as superoxide dismutase (SOD), catalase, Glutathione Peroxidase (GPx), Glutathione Reductase (GR), Glutathione−S−Transferase (GST) and nonenzymatic as reduced Glutathione (GSH) components which functions to neutralize free radicals. Increased oxidative stress amplifies the inflammatory response which accounts for disease severity. In case of cigarette smoking the release of exogenous and endogenous ROS and RNS by resident and recruited cells gets accumulated in the airways. This not only causes oxidative damage of DNA but is also responsible for lipids peroxidation, carbo and proteins denaturation thus mediate an array of downstream processes that contribute to the development and progression of pathogenesis of COPD^7^.

Presently, no specific treatments are available to halt the disease progression and suppress the lung inflammation effectively as still the pathophysiology of COPD needs to be explored^8^. New targets including antioxidants/redox modulator and candidates exhibiting anti − oxidant and anti − inflammatory potentiality are being explored as major strategies for the treatment and management of COPD. Recently, number of studies supports the effectiveness of natural products obtained from plants in attenuating and regulating inflammatory response and oxidative stress in many of the respiratory disease including COPD^9,10^. The relaxant effect of O. Basilicum extract was also observed in tracheal smooth muscles^11^. Rosmarinic acid a active component of Ocimum extract exerted strong anti − inflammatory and antioxidative effects and induced amelioration of lung injury comparable to that of dexamethasone in sensitised and asthmatic rats^12^. Rosmarinic acid can also stimulate the activity of antioxidant enzymes such as glutathione peroxidase, catalase, and superoxide dismutase^13^, and restore lung pathology in asthmatic rats^14^.

*Ocimum sanctum *(L). (Tulsi), popularly recognized as the “Queen of herbs” and “Elixir of life” is one of the holiest herbs belonging to Lamiaceae family and known for their aromatic components and pharmacological properties. Study supports the therapeutic potentiality of *Ocimum* as anti − inflammatory, antioxidative, antimicrobial, anticancerous, antidiabetic, antistress, antiviral, antifertility, antihelmintihic and immunomodulatory^15–17^. A recent GC − MS study in our lab has reported the presence of several phytocompounds including eugenol, cyclohexane, bicycle [7.2.0]undec − 4 − ene, 4,11,11 − trimethyl − 8 − methylene, Oxatricy − clo[8.2.0.0(4,6)]dodecane, 12 − trimethyl − 9 − methylene, tetracontane and phytol in majority in the hydroethanolic extract of *Ocimum* leaf extract^18^.

The present study aims to investigate the potential antioxidative activity of *Ocimum sanctum* leaf extract (OLE)against oxidative damage and lung injury induced by cigarette smoke in murine model. Further, an *insilico* study has been performed to support the potentiality of major phytocompounds (Eugenol, Cyclohexane and Caryophyllene) present in OLE extract against major oxidative enzymes (SOD, Catalse, GPx GR and GST).

## Results

### OLE inhibited the airway inflammation by suppressing total and differential cell count

**Figure 1:**
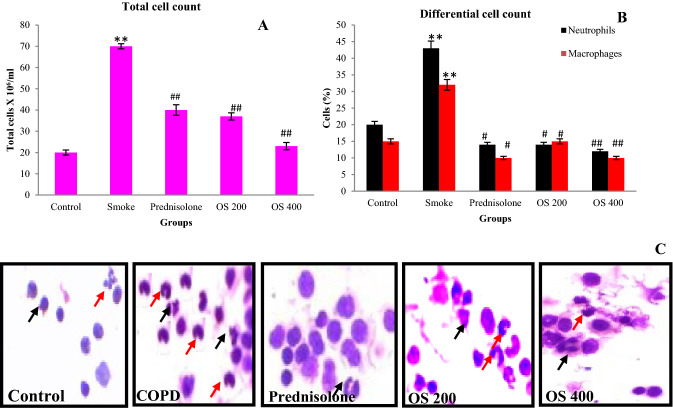
OLE inhibited the recruitment of immune cells to lungs as represented by Total cell count (**A**), Differential cell count (**B**), Cytospin slides (**C**): CS exposure leads increased leukocytes recruitment along with neutrophils and macrophages in lungs which were inhibited by the administration of OLE 200 mg/kg and 400 mg/kg doses. Values are expressed as mean ± SEM. Results were analysed statistically by one way ANNOVA followed by Turkey’s test. **p < 0.05 versus the normal group; ^#^p < 0.01 and ^##^p < 0.05 versus the COPD group. Red arrow represents macrophages; Black arrow represents neutrophils.

Total cells were counted in BALF to evaluate cellular infiltration into lungs. Remarkable increase in the number of total leukocyte recruitments was observed in COPD experimental mice as compared to the normal mice (Fig. 1A). The recruited cells mainly include the neutrophils (43%) and macrophages (32%) as counted on the cytospin slides (Fig. 1B,C). Pre − treatment with OLE downregulated the inflammation in the lungs (p < 0.05). This is supported by significant decrease in the accumulation of inflammatory cells accompanied with reduce neutrophils and macrophages.

### TOS and TAS regulation by OLE

**Figure 2:**
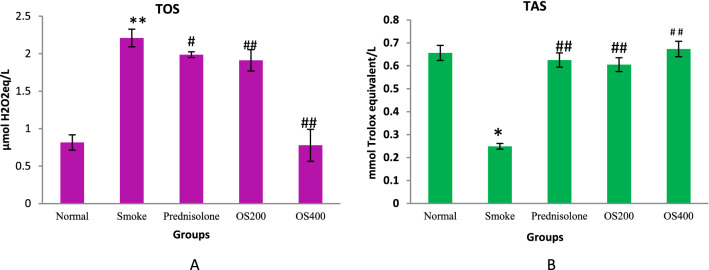
OLE modulated the total oxidant and antioxidant status in the lungs: CS exposure to mice leads an increased total oxidant (**A**) and decreased antioxidant status (**B**) in the lungs which were reversed by the administration of OLE with 200 mg/kg and 400 mg/kg. Values are expressed as mean ± SEM. Results were analysed statistically by one way ANNOVA followed by Turkey’s test. *p < 0.01 and **p < 0.05 versus the normal group; ^#^p < 0.01 and ^##^p < 0.05 versus the COPD group.

TOS was increased in COPD mice as compared to the normal mice whereas downregulation was found with OLE. There was an increase of 36.6% of total oxidants in the COPD mice as compared to normal mice (p < 0.05). The administration of OLE at 200 mg/kg and 400 mg/kg reduced the level of TOS to 8% and 59% respectively. TAS was declined in COPD mice to 42.5% while OLE at 200 mg/kg and 400 mg/kg upregulated the antioxidant status to 48% and 52.7% respectively (p < 0.01) (Fig. 2A,B).

### Protein and protein carbonyl content in BALF and lung

**Figure 3:**
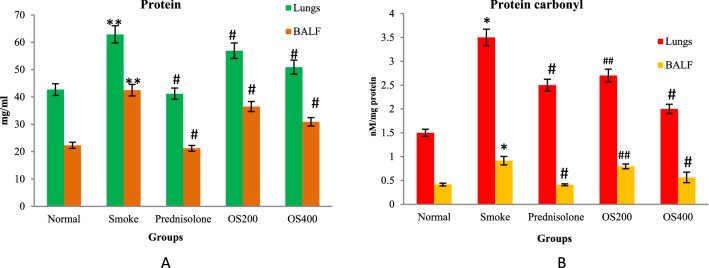
OLE suppressed protein and protein carbonyl content in CS exposed mice: Administration of OLE with 200 mg/kg and 400 mg/kg by i.p. route downregulated the protein concentration (**A**) and protein carbonyl content (**B**) which was upregulated by CS induction. Values are expressed as mean ± SEM. Results were analysed statistically by one way ANNOVA followed by Turkey’s test. *p < 0.01 and **p < 0.05 versus the normal group; ^#^p < 0.01 and ^##^p < 0.05 versus the COPD group.

The total protein and protein carbonyl concentration was found to be significantly elevated in BALF as well as lung homogenate of CS group as compared to the control group (p < 0.05). OLE treatment significantly attenuated the CS − induced enhanced total protein and protein carbonyl content in BALF as well as lung homogenate (p < 0.01 and p < 0.05) (Fig. 3A,B).

### Regulation of ROS, NO and MPO level

**Figure 4:**
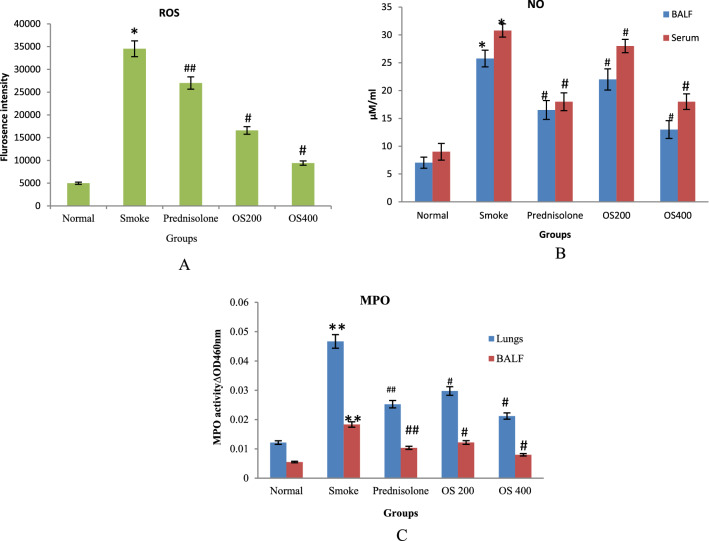
Effect of OLE on ROS (**A**), Nitrate (**B**) production and MPO level (**C**) in CS exposed mice: The increased level of ROS, nitrate and MPO in CS − induced mice was decreased with the pre − administration of 200 mg/kg and 400 mg/kg OLE. Values are expressed as mean ± SEM. Results were analysed statistically by one way ANNOVA followed by Turkey’s test. *p < 0.01 and **p < 0.05 versus the normal group; ^#^p < 0.01 and ^##^p < 0.05 versus the COPD group.

CS dramatically enhanced the ROS, NO and MPO level as compared with control group as observed in Fig. 4A–C respectively. On the contrary, pretreatment with OLE 400 mg/kg significantly reduced the ROS, NO and MPO content in samples, with greater effect than 200 mg/kg (p < 0.01). The administration, of prednisolone (1 mg/kg) remarkably decreased the level of ROS, NO and MPO but was found to be less effective compared to OLE 400 mg/kg.

### Modulation in lung antioxidant activity (SOD, Catalase, GPx, GR, GSH and GST)

**Figure 5:**
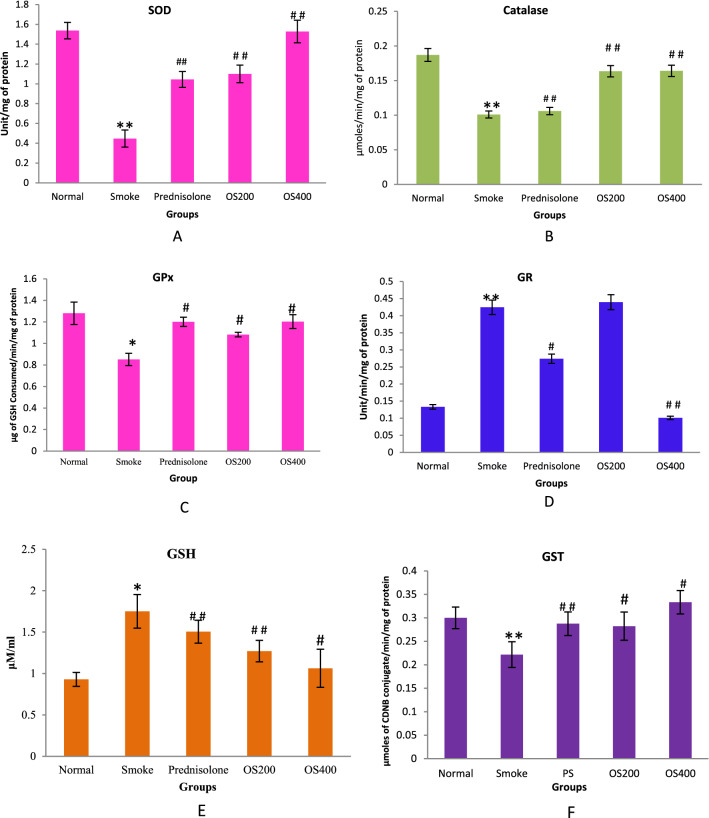
OLE reverted SOD (**A**), Catalase (**B**), GPx (**C**), GR (**D**), GSH (**E**) activity and GST content (**F**) in CS exposed mice: The modulated activity of SOD, catalase, GPx, GR and GST in CS − induced mice was reverted with the administration of OLE with 200 mg/kg and 400 mg/kg. The increased content of GSH in CS − induced mice was effectively decreased with the administration of OLE 200 mg/kg and 400 mg/kg. Values are expressed as mean ± SEM. Results were analysed statistically by one way ANNOVA followed by Turkey’s test. *p < 0.01 and **p < 0.05 versus the normal group; ^#^p < 0.01 and ^#^p < 0.05 versus the COPD group.

As depicted in Fig. 5A–C, the CS − induced COPD group exhibited significantly reduced activity of SOD (p < 0.05), Catalase (p < 0.05) and GPx (p < 0.01) as compared to the control group. However, administration of OLE significantly restored the activity of SOD (p < 0.05), Catalase (p < 0.05) and GPx to normal (p < 0.01). In contrast, the GR activity (p < 0.05) and GSH level (p < 0.01) were substantially elevated by CS exposure (p < 0.01). OLE treatment reduced GR activity and GSH level (Fig. 5D,E). OLE 200 mg/kg did not have any effect on GR activity. GST activity was also downregulated in CS exposed mice whereas upregulation in the activity was found with OLE treatment (Fig. 5F). OLE 400 mg/kg more effectively modulated the GST activity compared to 200 mg/kg. The antioxidative effect of OLE at 400 mg/kg was better than that of OLE at 200 mg/kg and standard drug.

### OLE inhibited MDA level

**Figure 6:**
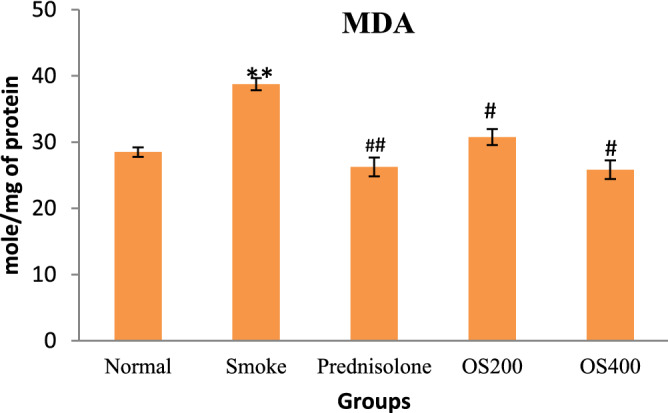
Effect of OLE on MDA level in CS exposed mice: OLE decreased the level of MDA in lung tissue in CS − induced mice where 400 mg/kg was significantly more effective. Values are expressed as mean ± SEM. Results were analysed statistically by one − way ANNOVA followed by Turkey’s test. **p < 0.05 versus the normal group; ^#^p < 0.01 and ^##^p < 0.05 versus the COPD group.

CS − induced COPD model showed adaptive increase in MDA level in the lungs as compared with the control group (p < 0.05). However, treatment with OLE (200 and 400 mg/kg) significantly decreased the levels of MDA content (p < 0.01). Moreover, treatment with prednisolone also significantly suppressed the elevated MDA content in lung homogenate (Fig. 6).

### OLE modulated TNF − α and IFN − γ cytokine levels

**Figure 7:**
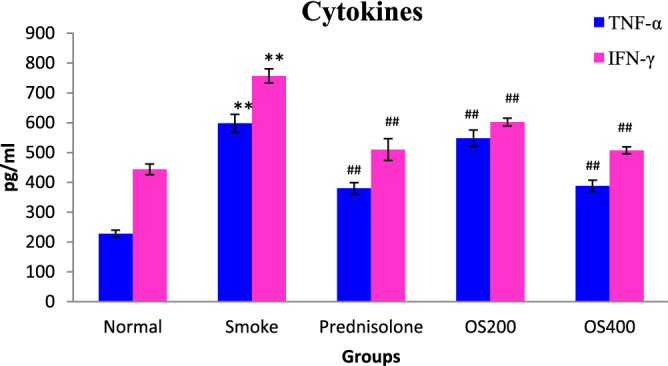
Effect of OLE on TNF − α and IFN − γ cytokine in CS exposed mice: OLE decreased the level of TNF − α and enhanced the level of IFN − γ in CS − induced mice. Results were analyzed statistically by one way ANNOVA followed by Turkey’s test. Values are expressed as mean ± SEM. **p < 0.05 versus the normal group; ^##^p < 0.05 versus the COPD group.

In BALF, the TNF − α and IFN − γ level was upregulated in CS − induced COPD model as compared to the control mice (p < 0.05). However, OLE significantly suppressed TNF − α and IFN − γ production (p < 0.05) compared to COPD mice (Fig. 7). Prednisolone also significantly reduced the level of both cytokines (p < 0.05).

### Emphysema and alveolar destruction

**Figure 8:**
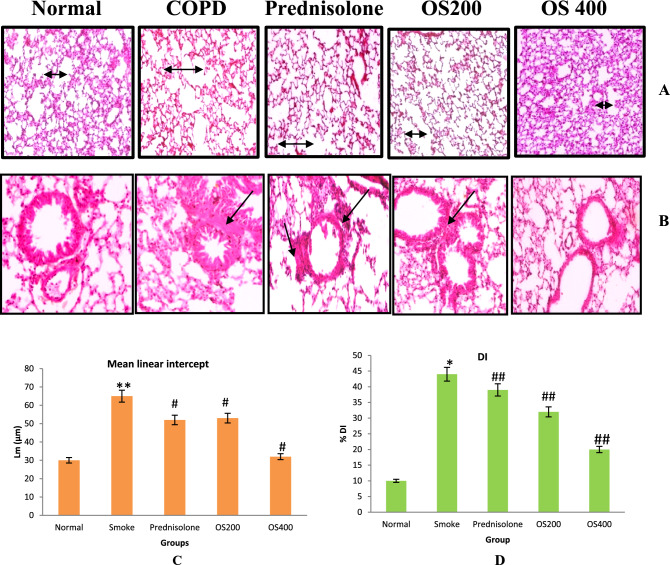
Micrograph of lung histopathology showing histological changes in lung segment stained with H&E after OLE treatment (**A,B**) Magnification X 100, Lm (**C**) and DI % (**D**). Control group showed no abnormalities and no destruction in the alveolar spaces and no peribronchiolar inflammation. COPD sections showed enlargement of the alveolar space with peribronchiolar inflammation. Prednisolone 1 mg/kg and OLE 200 mg/kg represent minor improvement in the airway enlargement and peribronchiolar inflammation while OLE 400 mg/kg effectively altered the alveolar spaces with no destruction and inflammation. The Lm (C) and DI (D) also exhibited improvement by OLE treatment in CS exposed mice. Values are expressed as mean ± SEM. Results were analysed statistically by one way ANNOVA followed by Turkey’s test. *p < 0.01 and **p < 0.05 versus the normal group; ^#^p < 0.01 and ^##^p < 0.05 versus the COPD group. Double headed arrow ( ↔) shows alveolar enlargement; single headed arrow ( →) shows peribronchiolar inflammation.

H&E staining was performed to evaluate the morphometric pathological changes in the lung tissue in terms of inflammation, emphysema and DI. As shown in Fig. 8A,B, the lung tissue mainly the alveolar spaces was significantly damaged as marked by enlarged airspaces and peribronchiolar inflammation was observed as a consequence of CS exposure. OLE and prednisolone modulated the alveolar destruction and peribronchiolar inflammation. L_m_ and % DI representing emphysematous changes, quantified in histological sections were significantly increased in CS − exposed mice compared to the control mice (p < 0.05 and p < 0.01) (Fig. 8C,D). However, lungs of OLE 400 mg/kg treated mice showed significantly lower L_m_ and % DI values compared to CS − exposed mice (p < 0.05).

### In silico study for antioxidant activity

**Figure 9:**
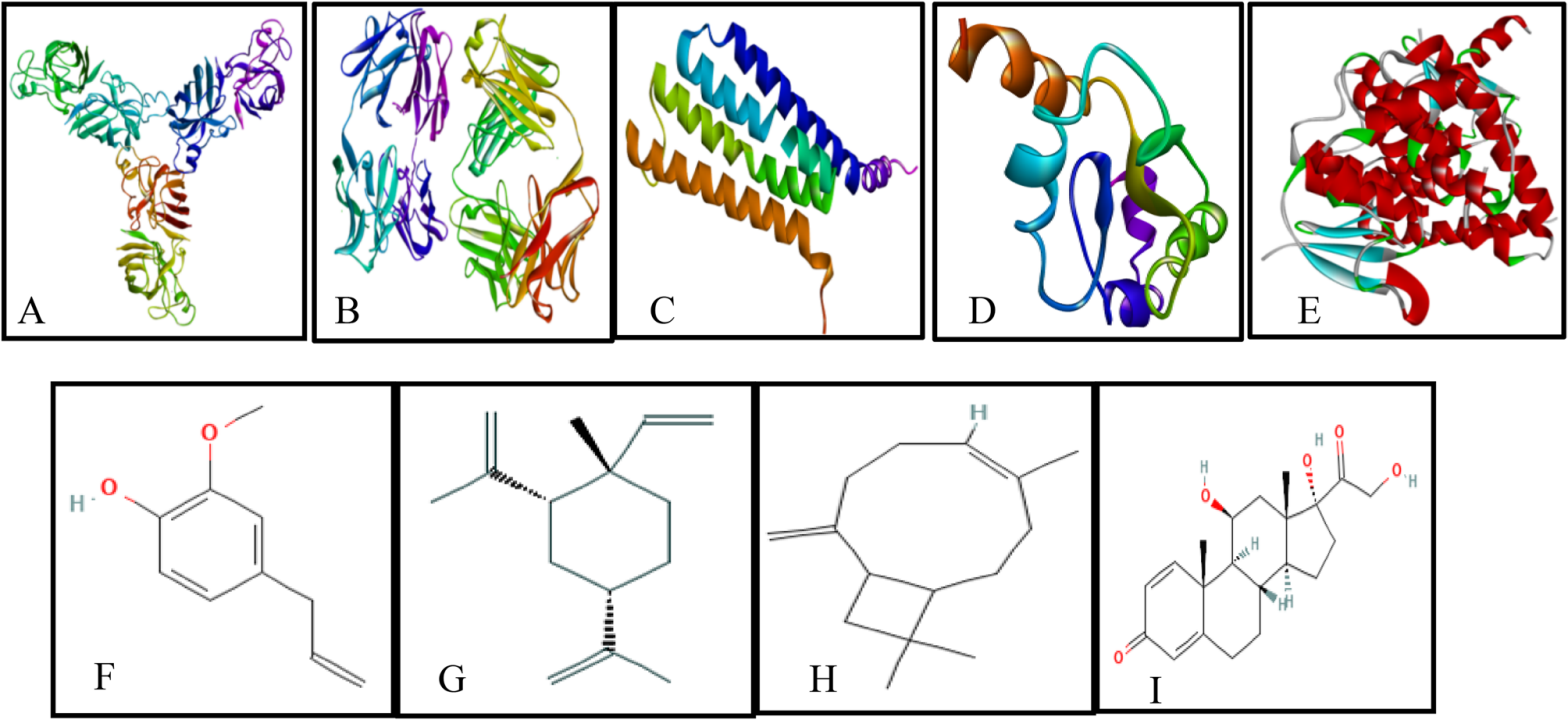
Cartoon representation of the oxidative stress enzymes and structure of major bioactive compounds of OLE extract: (**A**) Superoxide Dismutase; (**B**) Catalase; (**C**) Glutathione Peroxidase; (**D**) Glutathione Reductase; (**E**) Glutathione S Transferase; (F) Eugenol, (**G**) Cyclohexane (**H**) Caryophyllene; (**I**) Prednisolone.

**Figure 10:**
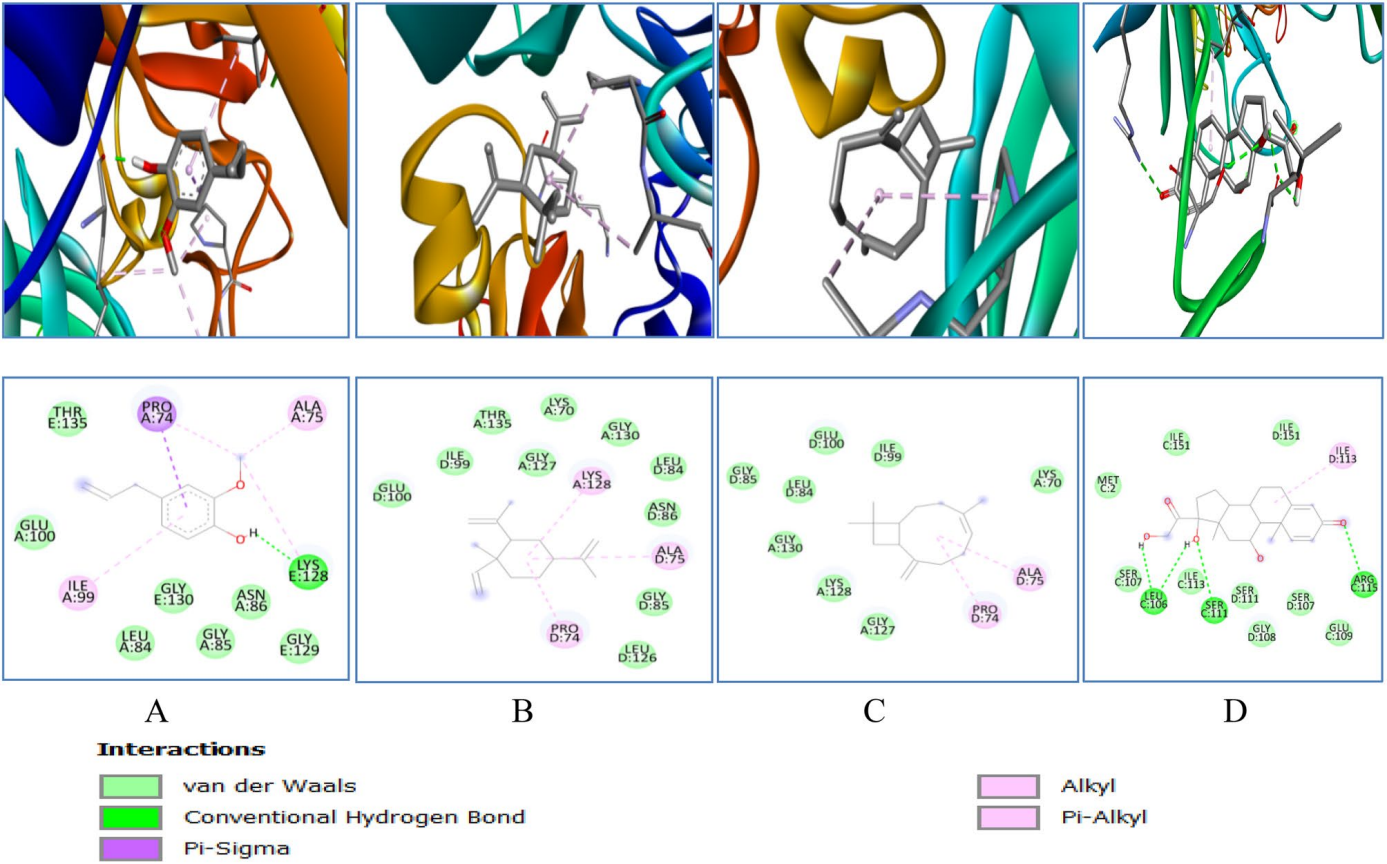
3D (to the upper panel) and 2D (to the lower panel) interaction diagrams of (**A**) Eugenol, (**B**) Cyclohexane (**C**) Caryophyllene, (**D**) Prednisolone with the crystal structure of SOD (PDB ID: 3GTT).

The protein structure of SOD, Catalase, GPx, GR and GST were obtained from PDB (Fig. 9A−E). 3D structure of eugenol, cyclohexane, caryophyllene and prednisolone (Fig. 9F−I) were obtained in SDF form from Pubchem for the study. The binding energies obtained from the docking of SOD with phytocompounds: eugenol, cyclohexane, caryophyllene and prednisolone were − 5.1, − 6.1, − 6.5 and − 7.8 kcal/mol respectively (Table 1). It formed different ligand–protein and 2D–3D interactions which includes conventional hydrogen bond with residue LEU106, SER111, ARG115,LYS 128, alkyl and π − alkyl interaction with ILE113, ALA 75, PRO 74 and ILE 99 respectively (Fig. 10).

**Table 1:** Molecular docking analysis of several phytocompounds against oxidative stress enzymes.

Oxidative stress enzymes	Ligands	Binding energy (ΔG)	No. of H − bond (drug enzyme)	Amino acid involve in interaction
SOD	Eugenol	− 5.1	1	LYSe128, ALAa75, PROa74, ILEa99
	Cyclohexane	− 6.1	0	LYSA128, ALAd75, PROd74
	Caryophyllene	− 6.5	0	ALAd75, PROd74
	Prednisolone	− 7.8	4	LEU106, SER111,ARG115,ILE113
CAT	Eugenol	− 6.4	0	TYRl34, TRPl93, TRPh33, ASNh35
	Cyclohexane	− 6.1	0	TYRl34, TRPh33, TRPl93
	Caryophyllene	− 8.2	0	TYRa34, TRPb33, TRPa93
	Prednisolone	− 7.8	1	ASN35, TYR34, TRP93, TRP33
GP	Eugenol	− 5.1	2	GLN25, PHE94, SER28, ALA29, VAL49, ALA98
	Cyclohexane	− 5.2	0	LEU87
	Caryophyllene	− 5.4	0	VAL11
	Prednisolone	− 6.5	1	ARG92, PHE85
GR	Eugenol	− 5.0	2	HIS101, VAL100, ARG32, ILE35, PHE96, ILE86
	Cyclohexane	− 5.6	0	ARG30, LEU34
	Caryophyllene	− 5.8	0	LEU34
	Prednisolone	− 7.0	2	ASN66, LEU121, VAL64
GST	Eugenol	− 5.9	3	CYS16, SER14, SER15, GLN111, ASN172, TYR11, ARG13, LEU116
	Cyclohexane	− 6.2	0	LEU113, VAL118, TRPa130, TRPb130
	Caryophyllene	− 6.1	0	TRPa130, TRPb130
	Prednisolone	− 7.7	2	SER72, GLN71,

Catalase binding energy was − 6.4, − 6.1, − 8.2 and − 7.8 kcal/mol with eugenol, cyclohexane, caryophyllene and prednisolone respectively (Table 1). Different 2D–3D interactions formed by phytocompounds, includes hydrogen bond, alkyl and π − alkyl interacting with residues ASN35, TYR 34, TRP 93, TRP 33 and ASN 35 (Fig. 11).

**Figure 11:**
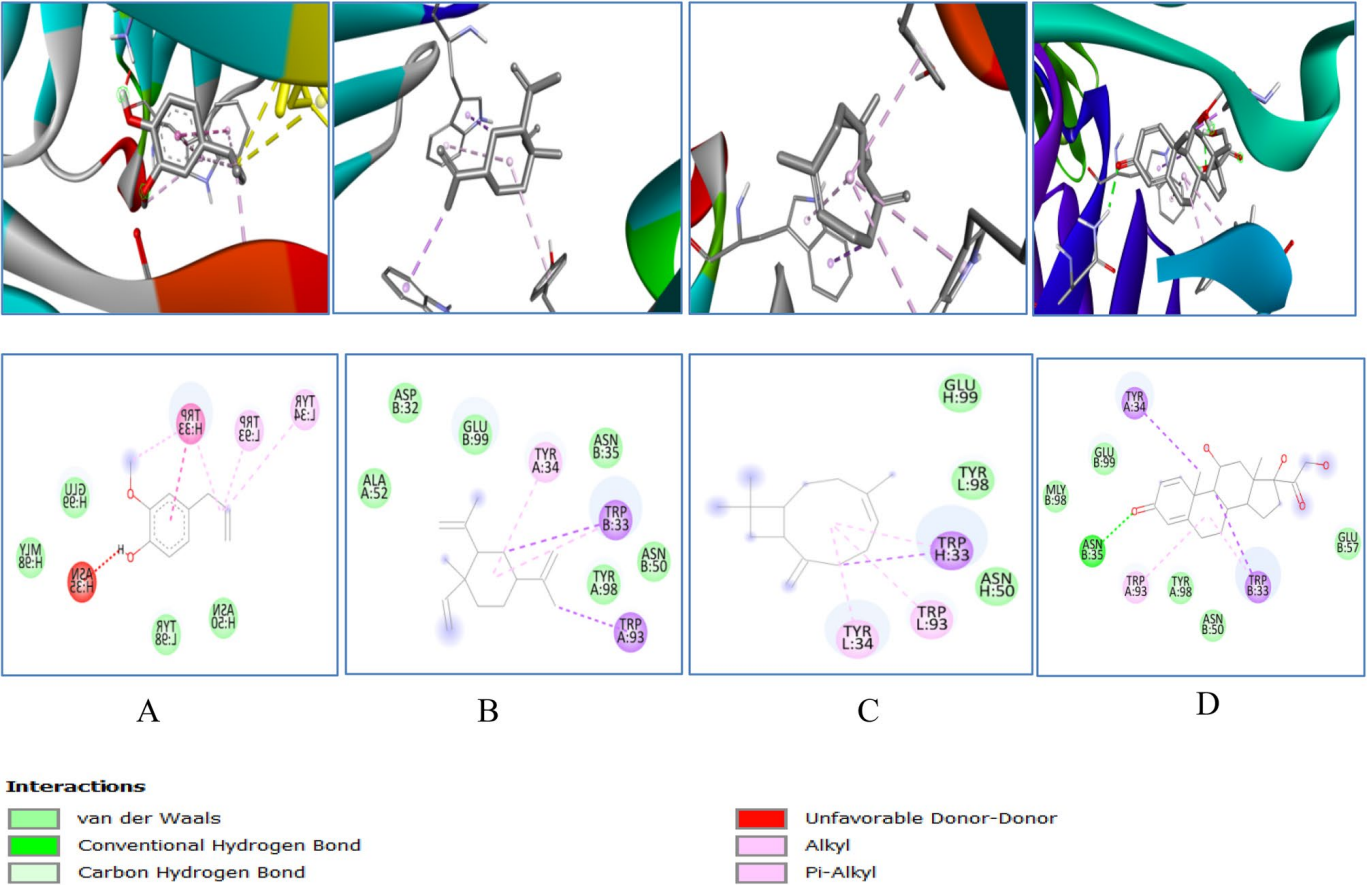
3D (to the upper panel) and 2D (to the lower panel) interaction diagrams of (**A**) Eugenol, (**B**) Cyclohexane (**C**) Caryophyllene (**D**) Prednisolone with the crystal structure of Catalase (PDB ID: 6NEX).

**Figure 12:**
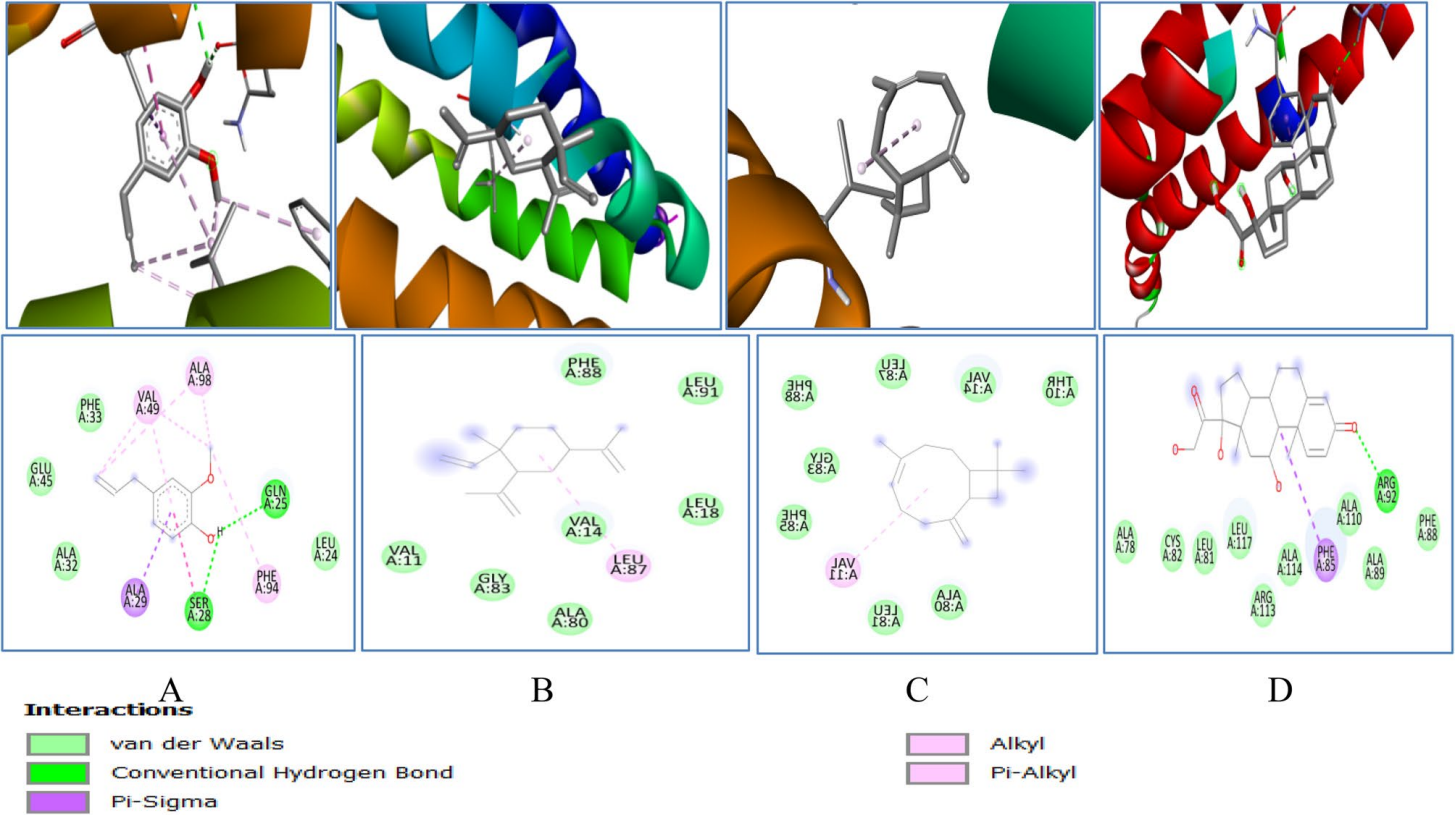
3D (to the upper panel) and 2D (to the lower panel) interaction diagrams of (A) Eugenol, (**B**) Cyclohexane (**C**) Caryophyllene (**D**) Prednisolone with the crystal structure of Glutathione Peroxidase (PDB ID: 4NTB).

The binding affinity of phytocompounds with GPx were − 5.1, − 5.2, − 5.4 and − 6.5 kcal/mol respectively for eugenol, cyclohexane, caryophyllene and prednisolone. The details of 2D − 3D interactions with the receptor protein are mentioned in Table 1. Interaction includes conventional hydrogen bonding with the residues ARG92, GLN 25 and SER 28, alkyl interaction with PHE85, PHE 94, ALA 29, VAL 49, ALA 98, LEU 87 and VAL 11 (Fig. 12).

**Figure 13:**
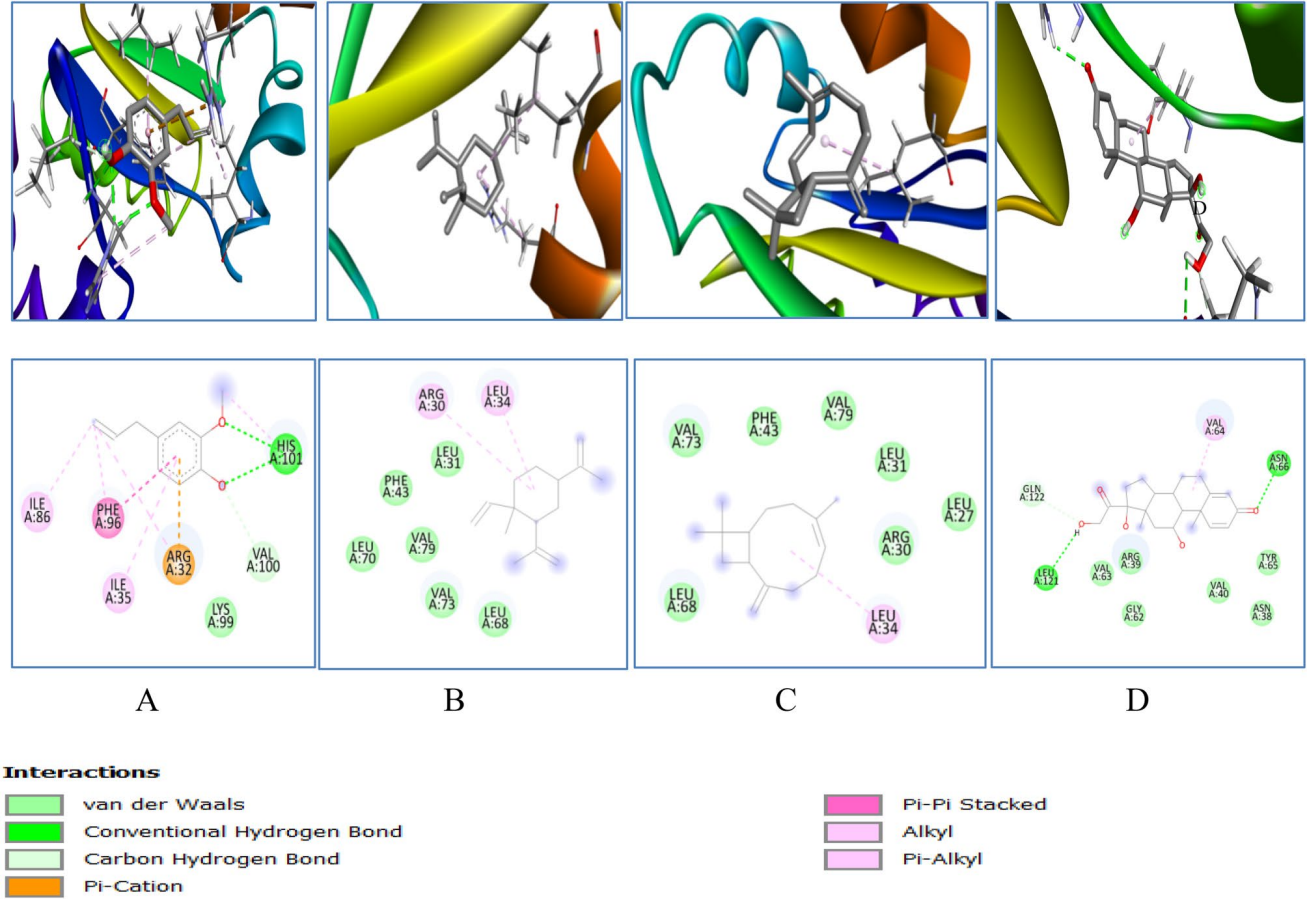
3D (to the upper panel) and 2D (to the lower panel) interaction diagrams of (**A**) Eugenol, (B) Cyclohexane (**C**) Caryophylleine (**D**) Prednisolone with the crystal structure of Glutathione Reductase (PDB ID: 2LV3).

GR binding energy was − 5.4, − 5.0, − 5.6 and − 7.0 kcal/mol with eugenol, cyclohexane, caryophyllene and prednisolone respectively. The different 2D–3D interactions involved were conventional hydrogen bonding with the residues LEU121, ASN66, HIS 101, alkyl interaction with VAL64, VAL 100, ILE 35, ILE 86, ARG 30, and LEU 34; π − π stacked with PHE 96, and π cation with ARG 32HIS 101, alkyl interaction with VAL 100, ILE 35, ILE 86, ARG 30, and LEU 34; Pi − Pi stacked with PHE 96, and π cation with ARG 32 (Fig. 13).

**Figure 14:**
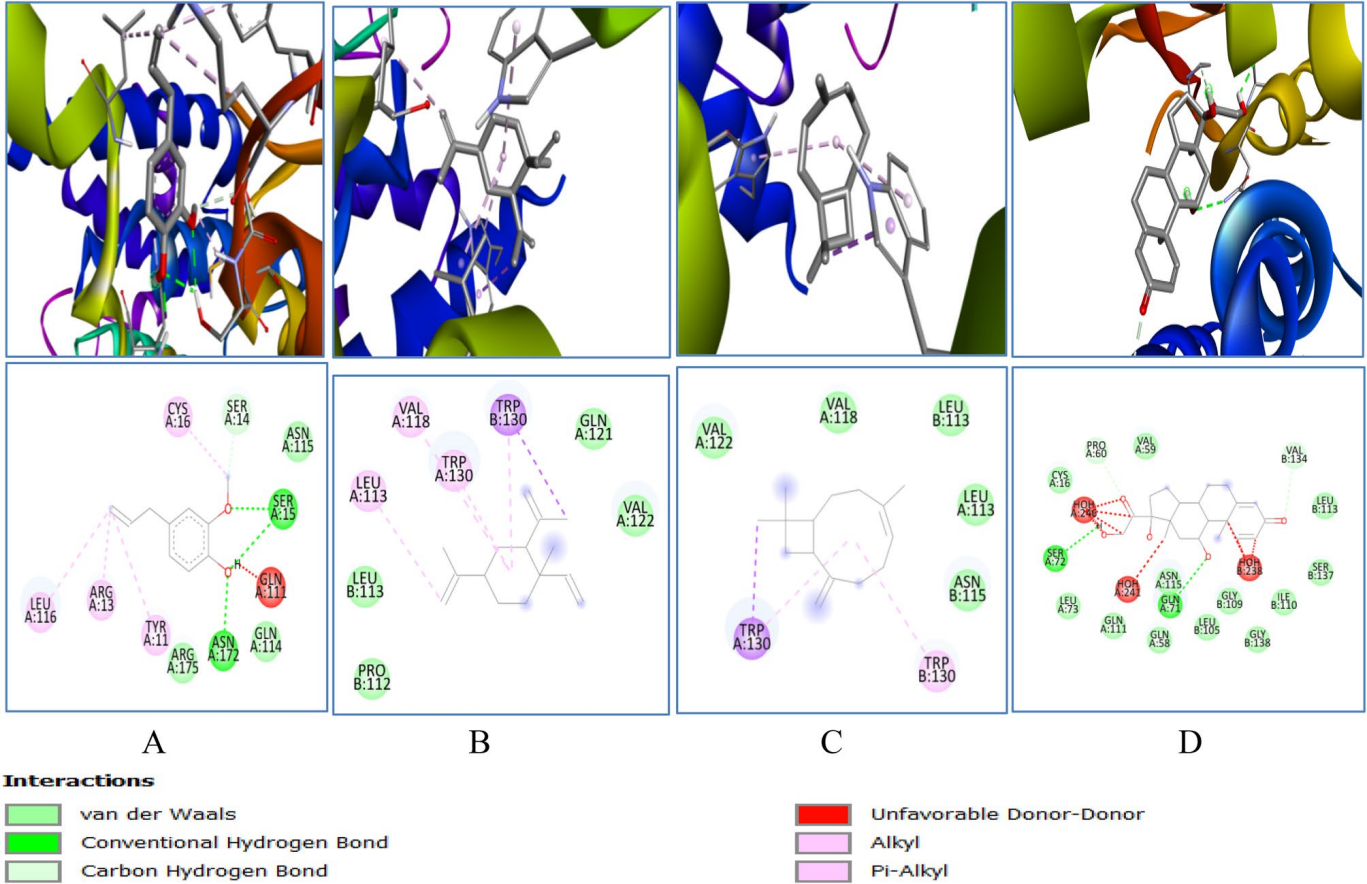
3D (to the upper panel) and 2D (to the lower panel) interaction diagrams of (**A**) Eugenol, (**B**) Cyclohexane (**C**) Caryophyllene (**D**) Prednisolone with the crystal structure of Glutathione − S − Transferase (PDB ID: 2CZ3).

Another enzyme GST was found to have binding energy of − 5.9, − 6.2, − 6.1 and − 7.7 kcal/mol with eugenol, cyclohexane, caryophyllene and prednisolone respectively (Table 1).It forms conventional hydrogen bond with the residue SER73, GLN71, SER 15 and ASN 172, alkyl and p − alkyl interaction with the residues − CYS16, SER14, GLN111, TYR11, ARG13, LEU116, LEU113, VAL118, TRP130 (Fig. 14).All the enzymes also formed several van der waals interactions with the remaining residues.

**Figure 15:**
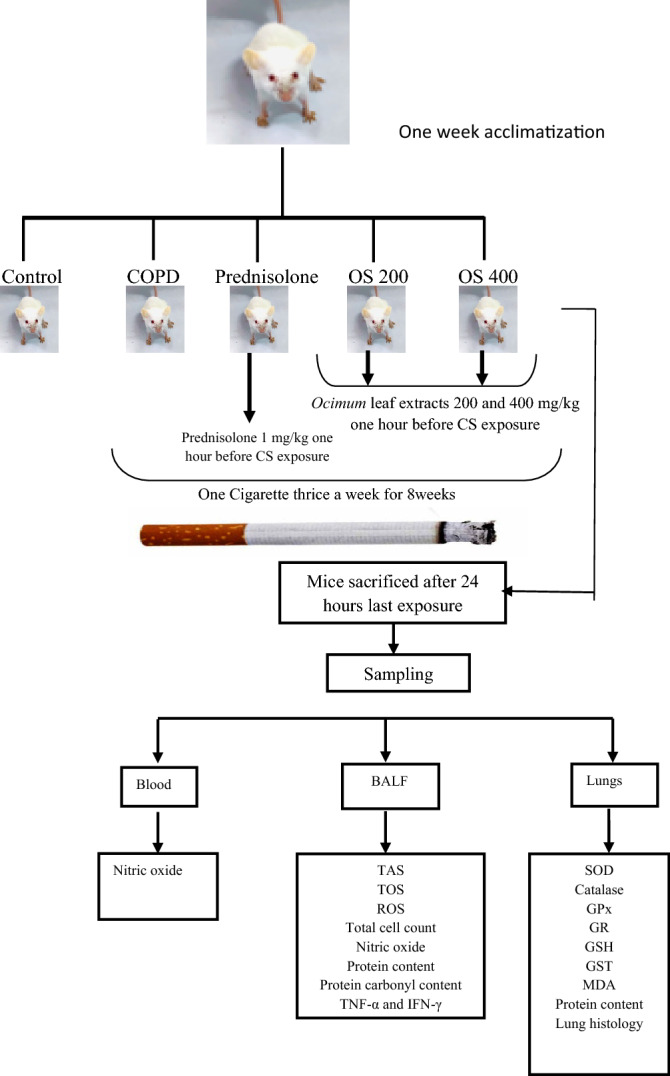
Schematic diagram of animal experimentation and protocol.

## Discussion

In the present investigation, the protective effect of OLE was studied in COPD induced by CS. For this, in vivo experiments were performed to demonsrate the downregulaion of inflammation and oxidative sress by OLE in murine model. The finding of in vivo experimens were further supported by in silico validation. CS has been used as an inducer to provoke the pathophysiology of COPD in mouse model and study the alterations in the airways as an imbalance of oxidant generation and antioxidant resistance and inflammation. Our finding provide evidences that OLE can be useful to suppress various pathological features associated with COPD. To the best of our knowledge this is the first study of its kind to report the potency of OLE in attenuating the inflammation and oxidative damage in CS − induced COPD mice by using both *invivo* and in silico approaches. Prednisolone, widely used as an anti − inflammatory drug, has been used as a positive control in investigating pulmonary dysfuntioning as a consequence of inflammation and oxiadative stress in COPD.

Numerous studies reported high content of toxic substance, potent oxidants and free radicals including quinone, hydroquinone, aldehyde, semi quinone and superoxide in cigeratte smoke^19^. Entrance of any irritant including CS into the respiratory tract alters the alveolar enviornment leading to epithelial cell damage and production of several cytokines and chemokines^20^. These cytokines and chemokines are responsible for inflammatory cell infiltration including neutrophils and macrophages and further their activation, leading to severe inflammatory response in airways^20^. The present investigation reports significant increase in infiltration and recruitment of immune cells (neutrophils and macrophages) in CS exposed mice where OLE treatment attenuated the inflammation by inhibiting the recruitment of cells including neutrophils and macrophages.

Recruited inflammatory cells are stated as rich source of endogenous ROS and RNS^21^. CS exposure is believed to drive ROS generation which distrupts macromolecules − DNA, protein and lipid thereby dysfunctioning many biochemical and physiological processes. Previous investigations proved ROS generation as a major marker for oxidant production trigerring inflammatory response by CS exposure. The protective effect of OLE was observed in ROS generation where enhanced production of ROS in CS − induced COPD was suppressed by OLE.

The levels of TAS and TOS are considered as marker of oxidative stress^22^. Most interestingly the present study also showed an upregulation in TOS and downregulation in Trolex equivalent antioxidant status (TEAS) in COPD mice which signifies imbalance of oxidants and antioxidants. The present result is also consistent with many of the previous studies where enhanced TOS and suppressed TAS in COPD subjects led to oxidants and antioxidants imbalance provoking to generate stress. OLE tends to regulate the TOS and TEAS levels suggesting its capability to maintain the balance of oxidant and antioxidant mechanism.

The prominent role of oxidative stress as a consequence of excess oxidants and reduced antioxidants defence in COPD pathophysiology is evident from several studies^18–20^. The generation of free radicals thereby depleting antioxidants generates an imbalance between oxidant production and antioxidant defense further initiating the phenomena of inflammation and oxidative stress^5,6,23^. It is well recognized that the endogenous antioxidant enzymatic profile constiting SOD, Catalase, GPx, GR and GST play an important role in free radical and peroxide metabolism protecting the cells against oxidant stress^22,24–27^. SOD and catalase, two crucial antioxidant enzyme functions simultaneously as oxide radical scavenger, thereby maintaining a balance between the production and scavenging of ROS. SOD functions to detoxifies the superoxide anion radicals by converting it into H_2_O_2_ and O_2_, while catalse decomposes H_2_O_2_ further to water and oxygen^28,29^. GPx and GR play a critical role in GSH productionwhich acts as a reducing substrate in the redox cycle facililating the reduction of H_2_O_2_ to H_2_O and O_2_ by GPx^30–32^. Further, GST functions to inactivate reactive electrophiles in coordination with GSH dependent mechanism and GR recycles the oxidized GSSG using NADPH as the reducing co − factor, thereby maintains appropriate intracellular GSH level in the cell^32^. In the present study, the COPD mice expressed decreased activity of SOD, CAT and GPx in lung tissues which may be responsible for increased oxidative stress. The decreased activity of SOD in COPD may be correlated with increased inflammation and ROS generation.OLE treatment modulated the antioxidant status in the tissue as evident from increased SOD, catalase and GPx. GR helps in generating GSH, hence the level of both GR and GSH was excerbated in COPD mice and suppressed in OLE treated mice.

On the other hand, GSH has also been reported to excerbate the level of NO and NO has been linked with increased neutrophilic inflammation^33^. In the present investigation, an increase in NO content was observed in COPD mice which was reduced in OLE treated group. The increased NO content might be due to increased neutrophilic inflammation and GSH level. Morever, GSH forms also conjugates with different electrophilic compounds, when the electrophile is very reactive, or more often through the action of GST. GST activity was also enhanced in COPD mice but declined with OLE treatment.

Peroxidase enzyme as MPO, released from activated neutrophil and macrophages is also considered as a potent source of ROS which catalyze the generation of hypohalous acids responsible for detrimental effect on the lungs amplifying inflammatory response^34,35^. The present study showed a sharp increase in MPO activity which was consistent with several prior investigations^36^. MPO activity in the lungs and BALF revealed inhibition by OLE treatment which might be correlated with the inhibition in ROS level also.

MDA, is considered a reliable marker of oxidative stress and has been observed as an index for lipid peroxidation further revealing the oxidative injury of cell membrane^37^. Studies indicates that increase in ROS level and decresed activity of SOD and catalase correlates with cellular membrane damage by inducing lipid peroxidation and the observation in the present study is similar to the previous findings^38^. Also, MDA content has been positively correlated with protein carbonyl content^39^. The present study showed an increase in MDA and protein carbonyl content in COPD mice which were impeded by OLE treatment.

Consistent with Th1 responses, TNF − *α* and IFN − *γ* representing synergistic effect have been reported to enhance COPD by elevating inflammation^40^. Both cytokines secreted by macrophages and T cells have been linked with the development of emphysema in mice^41^. The enhanced release of IFN − *γ* in COPD mice in the present study might be correlated with the increased release of TNF − α which may be further linked with the increased macrophages. OLE downregulated both cytokines, further supporting its role in regulating Th1 pathway at the local level.

Our study also demonstrates that OLE therapy significantly improved the histopathological changes in the lung tissue. As a form of lung tissue injury, alveoli with damaged parenchymal wall and peribronchiolar inflammaion appeared in CS − induced COPD mice in H&E stained section which was ameolirated after OLE treatment. Hence, OLE regulated many crucial factors of inflammation and oxidative stress thereby regulating pathological characters of COPD.

Computational tools along with experimental strategies have been of great value in modern drug design and development of novel promising compounds. Using computational tool model, we investigated possible inhibitory potential of major phytochemicalsof OLE (eugenol, cyclohexane and caryophyllene) on antioxidant defense system and compared its potentialiy with a sandard anti − inflammatory drug, prednisolone. The molecular docking, binding affinity with negative ΔG values and docking scores of eugenol, cyclohexane and caryophyllene in OLEwere comparable to prednisolone and revealed the capability of protein − targets (SOD, CAT, GPx, GR and GST: five key enzymes) binding of the antioxidant defense system. The docking results correlatesand supportes the in vivo antioxidants assay representing perfect interaction of protein − target.The present docking findings was found comparable wih initial findings obtained by Alminderej et al. where eugenol and caryophyllene (phytocompounds of extract of *Piper cubeba* L.) exhibited potential inhibitory effecton human protein target peroxiredoxin5^42^. Thus, it may besumarized that antioxidative tendency of OLE is due to the presence of different phytoconstituents which may be responsible for its potency.

The study also has certain limitations. The present study targets the effect of OLE on inflammatory and oxidative parameters. For the reason that remodeling is one of the characteristic feature of chronic COPD, further investigations needs to be performed on the remodeling parameters. Further to evaluate the mode of action of OLE, molecular signaling mechansism needs to be studied which may be supportive for the ongoing study.

## Conclusion

The present study supports the initial findings that airway inflammation and oxidative stress are connecting pathology in COPD. Our findings reflects the protective effect of OLE on the major indicators of oxidative stress, inflammation and lung injury. OLE 200 and 400 mg/kg body weight changed the oxidative/antioxidative imbalance by downregulating ROS, total oxidants, MDA level, NO, MPO and upregulating total antioxidant, SOD, catalase and GPx activities. Thus, OLE could be considered an effective therapeutic agent against CS − induced COPD. Further, studies are ongoing in our lab to explore the possible mechanistic action of OLE regulating pathophysiology of COPD before its clinical application.

## Methods

### Materials

2′,7′ − Dichlorofluorescin Diacetate (DCFDA), Vanadium chloride (III), Prednisolone (purity 99%), cetyltrimethylammonium bromide (CTAB), Xylenol orange and Trolox were purchased from Sigma − Aldrich (Burlington, USA). Bovine serum (BSA), *o − *dianisidine dihydrochloride (ODD), methionine, reduced Glutathione (GSH), oxidised Glutathione, nicotinamide adenine dinucleotide (NADH), hydroxylamine hydrochloride, 2,4 − dinitrophenylhydrazine and riboflavin were purchased from Sisco Research Laboratory (Mumbai, India). Sodium azide, 5, 5 − Dithiobis − 2 − Nitrobenzoic acid (DTNB) and 1 − chloro − 2,4 − di nitro benzene (CDNB) were purchased from Himedia (Maharashtra, India).

### Collection of plant material and preparation of leaf extract

*Ocimum sanctum* young and healthy leaves were collected and cultivated with relevant institutional national and international guidelines in Botanical Garden of Banaras Hindu University, Varanasi, India with permission of relevant authority. These were further identified, verified and authenticated by Prof N.K. Dubey, of the Department of Botany, Banaras Hindu University. A specimen voucher was submitted to the Department of Botany, with voucher number *Ocimum sanctum* (L) Lamia 2019/1.

Leaves extract was prepared by washing, air drying and crushing the leaves in blender to form fine dry powder. Hydroethanolic extract was prepared by Soxhlet apparatus using 5 g of crushed powered leaves in the Erlenmeyer flask containing 100 mL of 70% ethanol. The solution was applied to the apparatus for 24 h for complete extraction. The prepared extract was centrifuged, supernatant was filtered and further concentrated at 40 °C under reduced pressure using a rotary evaporator. The yield in respect of the dried extracts was 10%. The dried extract was stored at − 20 °C and reconstituted in double distilled water for experimental use.

### Experimental animals and grouping

Female balb/c mice (6–8 weeks old; 20–24 gms) used for the experiments were procured from the Central Drug Research Institute, Lucknow, India. The mice were acclimatized for a week under standard laboratory conditions (temperature 25 ± 3 °C; humidity 60 − 70%) and subjected on 12 h diurnal cycle. Mice were housed under pathogen − free conditions and fed with pellet diet (Platina Bharat Feeds and extraction Ltd., Indore M.P. − 451,010) and water ad libitum. Animal handling, maintenance and all the experimental protocols were approved by the Institutional Animal Ethical Committee, Institute of Medical Sciences, Banaras Hindu University with reference no. Dean/2015/CAEC/1410 and performed in accordance with the relevant guidelines and regulations.

Mice were randomly divided into five groups (8mice/group) as in Table 2. Group I was control mice and exposed to ambient air; Group II was COPD induced mice and exposed to CS as given in the protocol below; Group III was exposed to CS and treated with Prednisolone (1 mg/kg bw); Group IV was exposed to CS and treated with OLE extract (200 mg/kg bw) and Group V was exposed to CS and treated with OLE extract (400 mg/kg bw). Both extract and standard drug were administered intraperotonially one hour before cigarette smoke exposure.

**Table 2:** Grouping of animals.

Group	Inducer	Treatment	Route of administration and dose
Normal	Normal air	–	–
COPD	Cigarette smoke	–	–
Standard drug	Cigarette smoke	Prednisolone	i.p.; 1 mg/kg
OS 200	Cigarette smoke	Leaf extract of *O. sanctum*	i.p.; 200 mg/kg
OS 400	Cigarette smoke	Leaf extract of *O.sanctum*	i.p.; 400 mg/kg

### Development of COPD model by smoke induction and drug administration

Mice were exposed to CS to develop COPD in a closed chamber as per the protocol of Ghorani et al.^43^. The closed chamber consists of peristaltic pump, a fan to circulate the air into the chambers, a smoke generating chamber and a whole − body CS exposure chamber or inhalation chamber serially connected by silicone tubes. The smoke − exposed animals were subjected to two nonfiltered CS (Brand − Wills Navy cut manufactured and distributed by the ITC Limited Kolkata, India). Exposure to two CS was performed thrice a week till 8 weeks as represented in Fig. 9. The prepared leaf extract of *Ocimum* was administered at a dose of 200 mg/kg and 400 mg/kg. OLE was administered through intraperitoneal route in a total volume of 50 µl one hour before CS exposure. Dose determination (200 mg/kg and 400 mg/kg) for the present study was adapted from the previous studies^44,45^. Prednisolone was administered at a dose of 1 mg/kg bw through intraperitoneal route one hour before CS exposure. Twenty four hours after the last exposure, mice were euthanized. Broncheo Alveolar Lavage Fluid (BALF), lungs and blood were collected for study.

### Collection of sample

Mice were sacrificed by cervical dislocation 24 h after the last smoke exposure for collection of BALF, blood and the lungs. The trachea of the mice was cannulated and BALF was collected by injecting phosphate buffer saline (PBS). Briefly, the lung lumens were washed with 1 mL of ice chilled phosphate buffer three consecutive times and a total volume of 2.5 mL BALF was collected. Nearly 70% of the injected PBS was retrieved back in every wash. Collected BALF was centrifuged, and supernatant was used to analyse Nitric oxide (NO), Myeloperoxidase (MPO), protein and protein carbonyl content. BALF pellet cells were used to analyse total and differential cell count. Furthermore, the lungs were inflated with 10% Neutral buffer formalin (NBF), removed aseptically and preserved in 10% NBF for studying histological lung damage in alveolar spaces. The remaining lobes were homogenized in phosphate buffer, centrifuged at 3000 rpm and 4 °C and the supernatant was used to determine total oxidant and antioxidant status and antioxidant activities of SOD, Catalase, GPx, GR, GSH and GST. Lung lobes were also used for MPO and Malondialdehyde (MDA) study.

### Total and differential cell count

BALF suspension pellet was used for total cell count by trypan blue dye exclusion test. Briefly, the cells of the pellet were stained with trypan blue and counted in haemocytometer. The remaining BALF pellet cell suspension were cyto centrifuged at 800 rpm for 5 min on gelatin coated slides for differential count. The slides were air dried, fixed in methanol and stained with Geimsa stain. Immune cells were counted and enumerated on the basis of their nuclear morphology in a total number of 100 cells.

### Estimation of total oxidant status (TOS) and antioxidant status (TAS)

TOS was determined by the standardized protocol of Erel^46^. Briefly, reagent 1 (pH 1.75) was prepared by adding 150 μM of xylenol orange, 140 mM of NaCl and 1.35 M glycerol in 25 mM of sulphuric acid. Reagent 2 was prepared by adding 5 mM of ferrous ammonium sulphate and 10 mM of *o − *dianisidine dihydrochloride. The reaction was set up by adding 225 μl of reagent 1 and 35 μl of lung homogenate. First absorbance was measured at 560 nm and further the reagent 2 was added and after 3 − 4 min end point absorbance was observed in micro plate reader (Biotek, St. Louis, USA). Results of each of the samples were expressed as hydrogen peroxide equivalent per litre (μM H_2_O_2_ Equiv./L).

TAS was estimated by the established protocol of Erel^47^. Briefly reagent 1 (Clark and Lubs solution (75 mM, pH 1.8) was prepared by dissolving 75 mM of KCl, 10 mM of o − dianisidine dihydrochloride, 45 μM of Fe(NH_4_)_2_(SO_4_)_2_∙6H_2_O and 75 mM of reagent grade hydrochloric acid in 1000 mL of distilled water. Reagent 2 was prepared by mixing 7.5 mM hydrogen peroxide in 1000 mL of distilled water. Reaction mixture was setup by adding 200 µl of reagent 1 and 20 µl of lung homogenate. First absorbance was measured at 444 nm and further 10 µl of reagent 2 was added. Final absorbance was measured 3 − 4 min after adding reagent 2 in micro plate reader (Biotek, St. Louis, USA). Results of each sample were expressed in terms of millimolar Trolox equivalent per litre.

### Protein and protein carbonyl content

Total protein concentration in the BALF was measured according to the established protocol and bovine serum albumin (BSA) was used as a standard^48^. The protein concentrations in the samples were expressed as µg/mL. Protein carbonyl content was estimated in lung homogenate and BALF according to the protocol of Levine et al. where carbonylated protein was detected as derivative with 2,4 − Dinitrophenylhydrazine (DNPH) to produce achromophoric adduct which exhibited an extinction coefficient of 22,000 per mole per cm at 366 nm^49^. It was expressed as nM/mg of proteins.

### Estimation of reactive oxygen species (ROS)

BALF pellet cells were used for estimating ROS by the established protocol as previously described^50^. Cells were washed with cold PBS and counted with trypan blue for total viability. 1 × 10^5^ cells in 100 µl were plated and incubated in black plate with 100 µl of 10 mM of Dichlorodihydrofluorescein diacetate (DCFDA) for 45 min at 37 °C in the dark. 100 µl of PBS was added in blank group sample along with 100 µl of 10 mM of (DCFDA). After incubation, fluorescence intensity was measured by using spectrofluorometer at a wavelength of 490 excitation and 515 emission. Values of each sample are expressed in term of fluorescence intensity.

### Nitric oxide (NO) level

Nitric oxide was measured by Griess reagent in serum and BALF by the established protocol of Miranda et al.^51^. Briefly 100 µl of sample was mixed with 100 µl of 8 mg/mL of Vanadium III chloride (VCl_3_ for the release of nitric oxide. This was rapidly followed by the addition of Griess reagent which includes 50 µl of sulphamide (2% in distilled water) and 50 µl of N − (1 − Naphthyl) ethylenediamine (NED) (0.1% in 5% hydrochloric acid). Plate was incubated for 45 min to 1 h till pink colour was developed. O.D. was taken at 540 nm in a microplate reader (Biotek, St. Louis, USA). The concentration of the sample was measured against sodium nitrate used as standard. Values are expressed in µm/mL.

### Myeloperoxidase (MPO) activity estimation

MPO was measured in lung homogenate with slight modifications of established protocol^52^. Lung tissue homogenate (10%) was prepared in 50 mM phosphate buffer containing 0.5% cetyltrimethylammonium bromide (CTAB) and centrifuged at 12,000 rpm for 30 min. The supernatant along with the pellet were subjected to three times for freeze and thaw cycle and finally centrifuged at 12,000 rpm for 15 min. MPO assay was performed in 300 µl of total volume in 96 well microplate. In brief, 20 µl of the supernatant and BALF was mixed with 280 µl reaction mixture containing 0.167 mg/mL o − dianisidine dihydrochloride and 0.002% hydrogen peroxide in 50 mM phosphate buffer. The absorbance was measured at 460 nm for 20 min in a microplate reader. MPO activity was expressed as unit/mg of tissue and measured as a change in the absorbance within 20 min.

### Assessment of oxidative stress in lung homogenate

#### SOD activity

SOD activity was measured as previously described by Das et al.^53^. 10% lung homogenate was prepared in 50 mM phosphate buffer. Reaction mixture was prepared by mixing 1.14 mL 50 mM phosphate buffer (pH 7.4), 75 μl 20 mM α − methionine, 40 μl Triton X − 100, 75 μl 100 mM hydroxylamine hydrochloride and 100 μl 50 μM EDTA. 50 μl lung homogenate prepared in phosphate buffer was mixed with the reaction mixture and incubated for 5 min at 37 °C. Further, 80 μl of 50 μM riboflavin was added and incubated for 10 min in light inside a box coated with aluminium foil. Freshly prepared Griess reagent (1 mL) containing 1:1 solution of 0.1% NED and 1% sulphanilic acid in 5% orthophosphoric acid was added to reaction mixture. Absorbance of the mixture was taken by spectrophotometer (Aquamate, Thermo Scientific, Goteborg − Swedan) at 543 nm. SOD activity was expressed in per milligram of protein.

#### Catalase activity

Catalase activity was measured according to the previously described method of Aebi et al. with slight modification^54^. Reaction mixture was prepared by adding 10 µl of homogenate (prepared in 50 mM phosphate buffer), 490 µl distilled water, 1100 µl 50 mM phosphate buffer and 500 µl H_2_O_2_ (60 mM). Decrease in absorbance was observed for 5 min at 290 nm. Catalase was expressed in µmoles/min/mg of protein.

#### Glutathione peroxidase (GPx)

GPx was measured by the established protocol with slight modification^55^. Briefly, reaction mixture was prepared by adding 0.2 mL phosphate buffer (50 mM; pH 7.0), 0.1 mL sodium azide (10 mM), 0.2 mL lung homogenate (prepared in 50 mM phosphate buffer), 0.2 mL glutathione (4 mM) and 0.1 mL 25 mM H_2_O_2_. The tubes were incubated at 37 °C for 15 min, and the reaction was terminated by the adding 0.5 mL trichloroacetic acid (10%). To determine the residual glutathione, the reaction mixture was centrifuged at 1000 rpm for 10 min. After centrifugation, 1 mL supernatant was mixed with 1 mL DTNB (80 mg/mL in 1% sodium citrate). Absorbance was read at 412 nm spectrophotometrically (Aquamate, Thermo Scientific, Goteborg − Swedan). Results were expressed as μg of GSH consumed/mg protein.

#### Glutathione reductase (GR)

GR was estimated by the established protocol with minor modification^56^. Reaction mixture was prepared by adding 750 μl 0.2 M potassium phosphate buffer having 0.2 mM EDTA, 255 μl distilled water, 300 μl 2 mM NADPH, 75 μl oxidized glutathione (20 mM) and 20 μl lung homogenate. Absorbance was measured at 340 nm for 5 min in spectrophotometer. Decrease in absorbance indicates the activity of glutathione reductase. Results were expressed as units per mg of protein.

#### Reduced glutathione (GSH)

GSH was detected as per the previously discussed protocol^57^. 100 μl tissue homogenate, 600 μl reaction buffer containing 0.1 M sodium phosphate buffer (pH 7.0) and 1 mM EDTA were added. Further, 760 μl distilled water and 40 μl DTNB (0.04%) dissolved in 1% sodium tricitrate were added. The reaction mixture was incubated for 5 min and absorbance was read at 412 nm. Using the standard curve, GSH concentration for each unknown sample was determined and expressed as μM/mL.

#### Glutathione − S − transferase (GST)

GST activity was assayed by the standardized method of Macdonald et al., with certain modification^58^. Briefly, reaction mixture was prepared by adding 1 mL phosphate buffer (500 mM; pH 6.5), 100 µl 10% lung homogenate, 1.7 mL distilled water and 100 µl CDNB (30 mM in 95% ethanol). The mixture was then incubated at 37 °C for 10 min. After incubation, 100 µl reduced glutathione (30 mM) was added to the mixture. The mixture was centrifuged, and 1 mL of supernatant was mixed with 3 mL of reaction mixture (1.7 mL of phosphate buffer, 0.1 mL of CDNB and 1.2 mL of GSH). Absorbance was measured at 340 nm. Results were expressed as µmoles of CDNB conjugate/min/mg of protein.

#### Estimation of malondialdehyde (MDA)

MDA is the end product of major chain reactions leading to oxidation of fatty acids, and measurement of MDA is widely used for assessing lipid peroxidation. Lipid peroxidation was studied in the lungs by measuring MDA level in the form of thiobarbituric acid active substances with slight modifications^59^. In brief, 10% lung homogenate was prepared in potassium phosphate buffer (pH 7.4) and reaction mixture was prepared by adding 50 μl of homogenate, 50 μl of 8.1% Sodium dodecyl − sulphate (SDS), 375 μl of 20% acetic acid, 375 μl of 8.1% thiobarbituric acid and 150 μl distilled water. The reaction mixture was boiled for 1 h and cooled at room temperature to develop pink color, followed by addition of 250 μl distilled water and 1.25 mL butanol and pyridine (15:1). The reaction mixture was centrifuged at 2000 rpm for 10 min, separating into two layers. The absorbance of upper layer was taken at 532 nm, and MDA concentration was expressed in nanomoles per milligram of protein.

### Estimation of TNF − α and IFN − γ cytokine level

TNF − α and IFN − ƴ were measured in BALF supernatants with commercially available ELISA Kit (Biolegend, San Diego USA) as per the manufacturer’s instruction. Briefly, plates were coated with capture antibody and kept overnight at 4 °C. Plates were washed and primarily incubated for blocking for 1 h, then incubated with standards and samples for 2 h, further with detection antibody for 1 h, avidin − HRP for 30 min and finally incubated with TMB substrate solution in dark for 20 − 30 min till blue color appears. The reaction was stopped by adding 1 M H_2_SO_4_. The absorbance was taken at 570 nm and subtracted with absorbance at 450 nm. At every step plates were washed 3 − 4 times with Tris buffer saline tween 20 (TBST) and incubation was performed at room temperature. Using the standard curve, concentration for each sample was determined. The results are shown in pg/mL. Absorbance was read at 450 nm. Using the standard curve, concentration for each sample was determined in pg/mL (Supplementary Table).

### Lung emphysema and alveolar destruction by histological evaluation

For lung histology, the lung lobes were aseptically removed, fixed in 10% NBF, embedded in paraffin wax and 5 μm thin sections were sliced. Sections stained with haematoxylin and eosin (H&E) were used for light microscopy examination. Imaging was performed using an Olympus CX43 microscope (Tokyo, Japan). Sections with bronchioles were selected for peribronchiolar inflammation. Five full sections with 10 randomly selected fields per section were observed for evaluating emphysema (Mean Linear intercept; Lm) and destructive index (DI). Emphysema was observed as a measure of destruction of alveolar walls accompanied by damage lung parenchyma leading to enlarged alveolar spaces^60^. Quantification of air space was performed only in the sections without any cutting artifact. A grid with 40 points that were at the centre of hairline crosses was superimposed on the lung field. Structures lying under these points were classified as normal (N) or destroyed (D) alveolar spaces. DI was calculated as percentage of destroyed alveoli of all the alveoli counted per section^61^.

### In silico study to assess antioxidant property (SOD, Catalase, GPx, GR and GST) of major compound of OLE extract

#### Proteins and ligand structures

The crystallographic structure of oxidative stress enzymes (PDB IDs: SOD: 3GTT; CAT: 3NEX; Glutathione peroxidase: 4NTB; Glutathione Reductase: 2LV3; Glutathione S Transferase: 2CZ3) was retrieved from protein data bank (https://www.rcsb.org/) with a resolution: < 3 A֩ in protein data bank (PDB) format.

Three major phytocompounds (Eugenol, Cyclohexane and Caryophyllene) observed in the GC − MS analysis of extract were used for study. The study also comprised a standard anti − inflammatory drug i.e. prednisolone. The 3 − dimensional (3D) structures of phytocompounds and prednisolone were obtained from PubChem (https://pubchem.ncbi.nlm.nih.gov/), in spatial − data file (SDF) format. The ligands in SDF format were converted to Protein data bank (PDB) by using Open Babel (https://sourceforge.net/projects/openbabel/).

#### Molecular docking

Autodock 4.2 was used for protein optimisation by removing waterand other het atoms, and further adding a polar hydrogen group. Autodock 4.2 was supported by Autodock and MGL tools. Autogrid determined the native ligand positions on the binding site by arranging the grid coordinates (X, Y, and Z). Ligand tethering of the protein was performed by regulating the genetic algorithm (GA) parameters, using 10 runs of the GA criteria. The docking analyses were performed by both Autodock 4.2 and Biovia Discovery Studio 4.5^62^.

### Statistical analysis

The results are presented as mean ± standard error mean (SEM). Results were analyzed statistically between experimental group by one − way ANNOVA followed by Turkey’s post hoc test. Differences were evaluated for statistical significance and were considered significant at p < 0.01 and p < 0.05. All analysis was performed using statistical software SPSS 16.0.

### Ethics declarations

The experimental protocols were approved by the Institutional Animal Ethical Committee, Institute of Medical Sciences, Banaras Hindu University with letter no. is Dean/2015/CAEC/1410 and performed in accordance with the relevant guidelines and regulations.

### ARRIVE guidelines

The study is reported in accordance with ARRIVE guidelines.

## Supplementary Information

Supplementary Table 1.

## Data Availability

The data will be available from the corresponding author on reasonable request.

## Abbreviations

SOD: Superoxide dismutase
TOS: Total oxidant status
TAS: Total antioxidant status
ROS: Reactive oxygen species
MDA: Malondialdehyde
MPO: Myeloperoxidase
GSH: Reduced glutathione
SOD: Superoxide dismutase catalase
GPx: Glutathione peroxidase
GR: Glutathione reductase
CS: Cigarette smoke
BALF: Bronchoalveolar lavage fluid
MLI: Mean linear intercept
DI: Destruction index
NO: Nitric oxide
TNF − α: Tumour necrosis factor − α
IFN γ: Interferon γ
OLE: *Ocimum* leaf extract
