# Supplementary material for: Potential of hydroethanolic leaf extract of Ocimum sanctum in ameliorating redox status and lung injury in COPD: an in vivo and in silico study
Source: Sci Rep. 2023 Jan 20;13:1131. doi: 10.1038/s41598-023-27543-1 (PMC9860039; doi:10.1038/s41598-023-27543-1)
Supplement: Supplementary file 1 — Supplementary Table 1. [file 41598_2023_27543_MOESM1_ESM.docx]

**Potential of hydroethanolic leaf extract of *Ocimum sanctum* L in ameliorating redox status and lung injury in murine model of Chronic Obstructive Pulmonary Disease: An *in vivo* and *in silico* study**

Atul Srivastava^1^, Subhashini^2^, Vinita Pandey^2^, Vandana Yadav^2^, Sangita Singh^2^ and Ragini Srivastava^1*^

^1^Department of Biochemistry, Institute of Medical Sciences, Banaras Hindu University, Varanasi, India-221005

^2^ Department of Zoology, MMV, Banaras Hindu University, Varanasi 221005, India

*Corresponding author

Dr. Ragini Srivastava

Professor

Department of Biochemistry

Institute of Medical Sciences

Banaras Hindu University

Varanasi-221005, India

Email: [ragsriv@gmail.com](mailto:ragsriv@gmail.com)

Contact no: +919935174104

Supplementary data

Table: Showing different component present in GC-MS analysis of leaf extract of Ocimum sanctum

| Peak# | R. Time | Area | Area% | Name |
| --- | --- | --- | --- | --- |
| 1 | 7.843 | 141740 | 0.34 | BICYCLO[2.2.1]HEPTAN-2-OL, 1,7,7-TRIMETHYL-, (1S-ENDO)- |
| 2 | 11.085 | 2952103 | 7.06 | Eugenol |
| 3 | 11.343 | 231735 | .55 | Copaene |
| 4 | 11.433 | 485878 | 1.16 | Cyclohexane, 1-ethenyl-1-methyl-2,4-bis(1-methylethenyl)-, [1S-(1.alpha.,2.beta.,4.beta.)]- |
| 5 | 11.559 | 9027246 | 21.59 | Cyclohexane, 1-ethenyl-1-methyl-2,4-bis(1-methylethenyl)-, [1S-(1.alpha.,2.beta.,4.beta.)]- |
| 6 | 12.082 | 10130388 | 24.22 | BICYCLO[7.2.0]UNDEC-4-ENE, 4,11,11-TRIMETHYL-8-METHYLENE-, [1R-(1R*,4E,9S |
| 7 | 12.657 | 601275 | 1.44 | 1,4,8-CYCLOUNDECATRIENE, 2,6,6,9-TETRAMETHYL-, (E,E,E)- |
| 8 | 13.059 | 464226 | 1.11 | 1,6-CYCLODECADIENE, 1-METHYL-5-METHYLENE-8-(1-METHYLETHYL)-, [S-(E,E |
| 9 | 13.194 | 363823 | .87 | Naphthalene, decahydro-4a-methyl-1-methylene-7-(1-methylethenyl)-, [4aR-(4a.alpha.,7.alph |
| 10 | 13.302 | 470262 | 1.12 | ALPHA.-SELINENE |
| 11 | 13.476 | 155084 | 0.37 | CYCLOHEXANE, 1-ETHENYL-1-METHYL-2,4-BIS(1-METHYLETHENYL)-, [1S-(1.ALPHA.,2.BETA.,4.BETA.)]- $$ 2,4-DIISO, CYCLOPROP[E]AZULENE, Cycloheptane, 1,3,6,10-Cyclotetradecatetraene, |
| 12 | 14.627 | 3039100 | 7.00 | (-)-5-OXATRICYCLO[8.2.0.0(4,6)]DODECANE,,12-TRIMETHYL-9-METHYLENE-, [Caryophylele] |
| 13 | 15.040 | 140599 | 0.34 | (1R,3E,7E,11R)-1,5,5,8-Tetramethyl-12-oxabicyclo[9.1.0]dodeca-3,7-diene |
| 14 | 15.731 | 273776 | 0.65 | Isoaromadendrene epoxide |
| 15 | 16.446 | 244474 | 0.58 | 2-((2R,4aR,8aS)-4a-Methyl-8-methylenedecahydronaphthalen-2-yl)prop-2-en-1-ol |
| 16 | 16.698 | 737972 | 1.76 | 1,1,4,7-Tetramethyldecahydro-1H-cyclopropa[e]azulene-4,7-diol |
| 17 | 18.010 | 224757 | 0.54 | Neophytadiene |
| 18 | 19.717 | 1299736 | 3.11 | Ethyl 9-hexadecenoate |
| 19 | 19.975 | 797570 | 1.91 | HEXADECANOIC ACID, ETHYL ESTER |
| 20 | 20.317 | 149544 | 0.36 | 9-Hexadecenoic acid, (Z)-, TMS derivative |
| 21 | 20.556 | 473909 | 1.13 | Palmitic Acid, TMS derivative |
| 22 | 21.990 | 474438 | 1.13 | Ethyl Oleate |
| 23 | 22.059 | 229917 | 0.55 | (E)-9-Octadecenoic acid ethyl ester |
| 24 | 22.500 | 935411 | 2.24 | Phytol, acetate |
| 25 | 23.205 | 245778 | 0.59 | Glycidyl oleate |
| 26 | 23.419 | 229976 | 0.55 | Glycidyl palmitate |
| 27 | 26.013 | 422106 | 1.01 | 1,2-BENZENEDICARBOXYLIC ACID |
| 28 | 26.914 | 381217 | 0.91 | Eicosyl isopropyl ether, Hexadecyl isopropyl ether, Isopropyl tetradecyl ether |
| 29 | 28.594 | 169550 | 0.41 | 2-methyloctacosane |
| 30 | 30.587 | 619379 | 1.48 | Squalene |
| 31 | 31.684 | 211876 | 0.51 | Hexatriacontane |
| 32 | 32.902 | 259017 | 0.62 | Heptadecane, 3-methyl- |
| 33 | 34.040 | 617018 | 1.48 | STIGMAST-5-EN-3-OL, OLEAT |
| 34 | 34.314 | 422605 | 1.01 | Hexatriacontane |
| 35 | 38.041 | 1804127 | 4.31 | Tetracontane |
| 36 | 38.379 | 637256 | 1.52 | Stigmast-5-ene, 3.beta.-(trimethylsiloxy)-, (24S)- $$ Silane, trimethyl[[(3.beta.,24S)-stigmast-5-en-3-yl]oxy]- $$ Silane, trimethyl(stigma |
| 37 | 39.831 | 358191 | 0.86 | Dotriacontane, 1-iodo- |
| 38 | 40.520 | 338846 | 0.81 | Tetrapentacontane |
| 39 | 43.616 | 774787 | 1.85 | Hexatriacontane |
| 40 | 45.303 | 283938 | 0.68 | Phytyl palmitate |

Based on the above analysis three major compounds (Eugenol, Cyclohexane and Caryophyllene) having higher percentage area were selected for the in silico study.

**Authors Contribution**

Atul Srivastava – Searched literature, performed the experimental work.

Subhashini - Supervised the experimental work, performed the statistical analysis, contributed to drafting the manuscript.

Vinita Pandey – Searched literature, performed the experimental work.

Vandana Yadav- Searched literature, performed the experimental work.

Sangita Singh- Performed the in-silico study.

Ragini Srivastava – Designed the study and experimental work, research guidance, drafted the manuscript.
